# Long-Term Effects of Maternal Deprivation on the Volume of Dopaminergic Nuclei and Number of Dopaminergic Neurons in Substantia Nigra and Ventral Tegmental Area in Rats

**DOI:** 10.3389/fnana.2020.578900

**Published:** 2020-10-23

**Authors:** Slobodan Kapor, Milan Aksić, Laslo Puškaš, Marin Jukić, Joko Poleksić, Filip Milosavljević, Suncica Bjelica, Branislav Filipović

**Affiliations:** ^1^School of Medicine, Institute of Anatomy “Niko Miljanić”, University of Belgrade, Belgrade, Serbia; ^2^Department of Physiology, Faculty of Pharmacy, University of Belgrade, Belgrade, Serbia; ^3^Pharmacogenetics Section, Department of Physiology and Pharmacology, Karolinska Institutet, Stockholm, Sweden; ^4^Group for Molecular Oncology, Institute for Medical Research, University of Belgrade, Belgrade, Serbia; ^5^Department of Hematology, Clinical Center “Dragiša Mišović”, Belgrade, Serbia

**Keywords:** early life stress, dopamine, substantia nigra, ventral tegmental area, glucocorticoids

## Abstract

Early life adversities leave long-lasting structural and functional consequences on the brain, which may persist later in life. Dopamine is a neurotransmitter that is extremely important in mood and motor control. The aim of this study was to investigate the effect of maternal deprivation during the ninth postnatal day on the volume of dopaminergic nuclei and the number of dopaminergic neurons in adolescence and adulthood. Maternally deprived and control Wistar rats were sacrificed on postnatal day 35 or 60, and the dopaminergic neurons were stained in coronal histological sections of ventral midbrain with the tyrosine hydroxylase antibody. The volume of dopaminergic nuclei and the number of dopaminergic neurons in the substantia nigra (SN) and ventral tegmental area (VTA) were analyzed in three representative coordinates. Maternal deprivation caused weight loss on postnatal day 21 (weaning) and corticosterone blood level elevation on postnatal days 35 and 60 in stressed compared to control rats. In maternally deprived animals, the volumes of SN and VTA were increased compared to the controls. This increase was accompanied by an elevation in the number of dopaminergic neurons in both nuclei. Altogether, based on somatic and corticosterone level measurements, maternal deprivation represents a substantial adversity, and the phenotype it causes in adulthood includes increased volume of the dopaminergic nuclei and number of dopaminergic neurons.

## Introduction

Development of the nervous system plays a pivotal role in the establishment of healthy adult brain, while stressful events in critical neurodevelopmental periods may cause abberations and maladaptive phenotypes, such as schizophrenia and other neurocognitive disorders (Murray et al., 1992; Waddington et al., 1997). In support of this, maternal deprivation (MD) has been proposed as an animal model for studies of certain neuropsychiatric disorders, including schizophrenia (Ellenbroek and Cools, 2002). Maternal deprivation is an animal model in which rat pups are suddenly separated from their mothers for a defined period of time (Husum and Mathe, 2002; Aksic et al., 2013). Early traumatic experiences are postulated to cause long-term neural changes which are an integral part of the etiology of numerous psychiatric disorders (Llorente et al., 2012). Ellenbroek and Cools (2002) have shown that early maternal deprivation induces schizophrenia-like phenotypes. In experimental animals, early life stress caused by maternal deprivation of pups during a critical period of development is known as the stress hyporesponsive period (SHRP) (Chocyk et al., 2011). The SHRP for rats occurs between postnatal days 4 and 14, and it is a period of time during which pup–mother interactions, such as licking, grooming, and arched-back nursing, suppress the basal and stress-induced levels of glucocorticoids (de Kloet et al., 2005). One of the main biological functions of the SHRP is to protect the developing brain from fluctuations of glucocorticoid levels (Champagne et al., 2009). Glucocorticoids profoundly affect brain development as they are involved in neurogenesis, gliogenesis, myelination, differentiation, and apoptosis (Almeida et al., 2000; Champagne et al., 2009). Adversities during SHRP can profoundly affect the neuronal functioning later in life, and the timing has been shown to be crucial for the development of various phenotypes. It has been previously shown that early maternal deprivation can disrupt normal salience in adult rats and that this phenotype is dependent on the day of deprivation (Ellenbroek and Cools, 2002). In particular, prepulse inhibition was disrupted when deprivation took place on postnatal day 9 (P9), but it remained intact when the rats were deprived on P13 (Ellenbroek et al., 1998; Ellenbroek and Cools, 2002), meaning that prepulse inhibition was most severely affected when deprivation took place around P6–P9, right in the middle of SHRP (Ellenbroek and Cools, 2002).

The substantia nigra (SN) and the ventral tegmental area (VTA) are the principal dopaminergic nuclei with projections throughout the brain and which control various processes. The enrichment of dopaminergic innervation continues to increase until postnatal day 60 in rats, whereas after that point, the density and topography of the dopaminergic afferents remain relatively constant (Suri et al., 2015). Profound changes in the dopaminergic system morphology and signaling caused by early life stress have been observed in a series of previous findings; in particular, maternal deprivation increased the circulating level of dopamine in the rat brain (Huppertz-Kessler et al., 2012) and increased the number of tyrosine hydroxylase positive neurons in the SN (Chocyk et al., 2011) and VTA (Jahng et al., 2010) later in life. In addition, a short period of maternal deprivation increased the expressions of Drd1 and Drd2 throughout many regions of the adult brain (Ploj et al., 2003; Brenhouse et al., 2013; Li et al., 2013). However, relatively little is known regarding the consequences of early life stress on the dopaminergic system morphology during adolescence and adulthood.

Previous studies have shown an important role for glucocorticoids, primarily dexamethasone, which mimic the effects of stress and which can alter the cytoarchitecture and number of dopaminergic cells in the SN and VTA in adult rats when administrated during the prenatal and early postnatal periods (McArthur et al., 2005). Previous morphometric microscopy studies revealed alterations in the neuronal density of the limbic system and temporal and frontal cortices (Arnold and Trojanowski, 1996). During the first 2 weeks of postnatal development, the dopaminergic neurons are particularly sensitive to adversities (Chocyk et al., 2011); therefore, the aim of this study was to investigate the effect of maternal deprivation conducted on the ninth postnatal day on the volume of dopaminergic nuclei and the number dopaminergic neurons in the dopaminergic nuclei in adolescent and adult rats.

## Materials and Methods

### Animals and Procedures

Male and four nulliparous female Wistar rats (3 months old) were put together in standard Plexiglass cages with normal embedding (26 × 42 × 15 cm) in a temperature-controlled room (23 ± 1°C). The rats were on a standard 12-h light/dark cycle with lights on from 7:00 a.m. to 7:00 p.m., with freely available water and food. Two weeks later, the males (one per each group) were removed, and pregnant female (one per each group) rats were checked twice daily for a potential delivery. The day of delivery was noted as postnatal day 0 (P0). On P9, two litters were weighed and subjected to the maternal deprivation (MD) procedure according to the previously published protocol (Ellenbroek and Cools, 2000; Roceri et al., 2002; Aksic et al., 2013; Aleksic et al., 2016). Briefly, the mothers were removed from the litter at 10:00 a.m., after which the pups were weighed and returned to their home cage. They remained in their home cage at room temperature for 24 h. This meant that the pups also remained without access to food during this period. The mothers of the control litters (two groups) were very briefly (3 min) removed from their home cages. All litters were later left undisturbed, except for the routine cleaning of the cages twice per week, until P21, when the litters were weaned and classified according to gender; at this point, the animals were weighed. For this experiment, 20 animals were divided into four groups, out of which two groups (five control and five MD rats) were sacrificed on P35, which corresponds to periadolescence. The other two groups (five control and five MD rats) were sacrificed at the period of young adulthood (P60). All efforts were made to minimize animal suffering and reduce the number of animals used in the study. All experimental procedures were in compliance with the EEC Directive (2010/63/EU) on the protection of animals used for experimental and other scientific purposes and were approved by the Ethical Committee for the Use of Laboratory Animals of the School of Medicine, University of Belgrade (approval no. 323-07-01245/2014-05/2).

### Tissue Processing

The rats were deeply anesthetized and transcardially perfused, starting with a vascular rinse until the liver was discolored (200 ml of 0.9% saline, perfusion speed of 40 ml/min), followed by a 4% paraformaldehyde solution in 0.1 M phosphate-buffered saline (PBS; 200 ml, 100 ml at 40 ml/min and then 30 ml/min), and finally with the 10% sucrose solution in 0.1 M PBS (200 ml, 30 ml/min). The animals’ brains were extracted and cleared of the meninges and blood vessels and then immersed in 4% paraformaldehyde solution overnight. On the next day, the brains were moved to the 30% sucrose solution for several days until they sunk and then were flash frozen. The brains were cut in a coronal plane into 40-μm-thick sections using a cryostat (Leica Instruments, Nußloch, Germany). Free-floating sections were stained, mounted, and coverslipped with DPX (Sigma) and then examined under an Olympus CH2 (Japan) microscope equipped with a camera.

Immunohistochemistry was performed under the same conditions for all experimental samples, and the volume of the dopaminergic nuclei as well as the number of dopaminergic neurons were quantified based on tyrosine hydroxylase (TH) positively immunostained (TH +) cells in the substantia nigra pars reticulata (SNpr), substantia nigra pars compacta (SNpc), and in the ventral tegmental area (VTA). Brain sections were initially thoroughly rinsed with 0.1 M PBS, pH 7.4. The endogenous peroxidase activity was neutralized with 3% hydrogen peroxide/10% methanol for 15 min, and non-specific binding was prevented by 60 min incubation in 5% normal donkey serum (D9663, Sigma-Aldrich, United States)/0.1 M PBS at room temperature. The sections were further incubated for 48 h at 4°C with primary mouse monoclonal anti-TH antibody (dilution 1:1,000, T2928, Sigma-Aldrich, United States) in blocking solution (0.5% Triton X-100 in 0.1 M PBS) and subsequently for 90 min in donkey anti-mouse IgG-HRP (dilution 1:50, sc-2318, Santa Cruz, United States). Between each immunolabeling step, the sections were washed in fresh 0.1 M PBS (3 × 5 min). Immunoreactive signals were visualized with a diaminobenzidine solution [1% 3,3′-diaminobenzidine (11208, Acros Organics)/0.3% hydrogen peroxide/0.1 M PBS]. All sections were finally mounted on slides, dehydrated in a series of solutions with increasing ethanol concentrations (70, 96%, and 100% ethanol; Zorka Pharma, RS), placed in a clearing agent (xylene; Zorka Pharma, RS), mounted with DPX (Sigma-Aldrich, United States), and coverslipped.

### Serum Corticosterone Assay

After the sacrifice of animals on P35 and P60, blood was collected from the rats in both the MD and control groups by cardiac puncture. After the blood was harvested, it was allowed to clot for 1 h at room temperature and centrifuged at 2,000 rpm for 10 min at 4°C to obtain the serum. The serum samples were collected into ice-cooled tubes and stored at −20°C until the corticosterone assay. Corticosterone concentration was measured in 1:10 diluted serum samples using a commercially available enzyme immunoassay (EIA) kit (IDS; EURL Paris, France). Briefly, 100 μl of the diluted serum or corticosterone standards was added to the wells of an antibody-coated microtiter plate. Then, 100 μl of the enzyme conjugate was added to each well of the plate and incubated for 24 h at 4°C. After the plate was washed three times with the wash solution, 200 μl of tetramethylbenzidine (TMB) substrate was added to each well and incubated for 30 min at room temperature. The absorbance was read at 450 nm using a microplate reader within 30 min of adding the stop solution. Each sample was run in duplicate and the concentration of corticosterone was determined by using the calibration curve based on the concentrations of the external standards.

### Volumetric Measurements of Dopaminergic Nuclei

All the tissue samples of the control and experimental brains (three sections per rat) were grouped into three defined stereotaxic ranges of the overall SNpr, SNpc, and VTA rostro-caudal dimensions. Namely, the volume of each structure was approximated by the surface area in three 40-μm-thick coronal sections per brain, representing the overall rostro-caudal dimension of SNpr, SNpr, and VTA in three defined stereotaxic ranges (4.60–5.10 mm, 5.20–5.70 mm, and 5.80–6.30 mm caudally from bregma). The whole SNpr, SNpc, and VTA were delineated in three stereotaxic ranges and the area was measured from the micrographs using ImageJ 1.46 software. All measurements were performed on both sides of each brain in a defined stereotaxic range, after which the mean value of the measured sides was used as a result for the statistical analysis of each brain. The volumes of the whole SNpr, SNpc, and VTA were approximated by the surface area in three coordinates according to Cavalieri’s principle (Chocyk et al., 2011), i.e., [Area 1 (left side of the section) + Area 2 (right side of the section)]/2 × 0.04 (thickness of the section/1,000).

### Image Acquisition and Quantitative Analysis of Tyrosine Hydroxylase (TH +) Cells

Images were taken using an Olympus CH2 (Japan) microscope and analyzed in ImageJ 1.46 software. Tyrosine hydroxylase-positive cells were counted in stereological sections of the rat brains through the overall rostro-caudal dimension of SNpr, SNpc, and VTA in three defined stereotaxic ranges (4.60–5.10 mm, 5.20–5.70 mm, and 5.80–6.30 mm caudally from bregma). All counts were performed on both sides of each brain in a defined stereotaxic range, after which the mean value of the measured sides was taken for each brain as results for statistical analysis.

### Statistical Analysis

All numerical data are presented as group mean values with standard errors of the mean (SEM). All statistical analyses were done in the IBM SPSS 11.0 software using Student’s *t* test. The differences were considered significant if the *p*-value was lower than 0.05.

## Results

**FIGURE 1:**
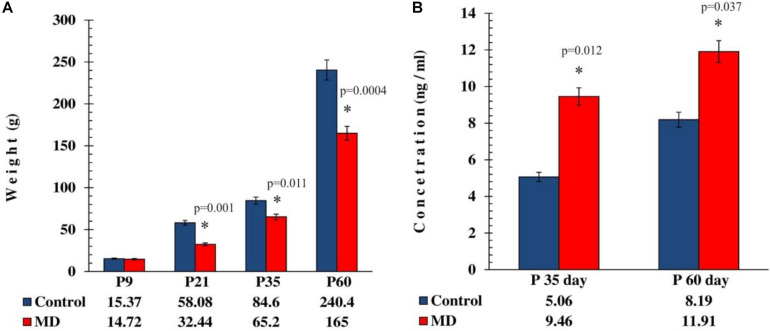
Effect of maternal deprivation on the animals’ weight at weaning age and on the corticosterone levels in adolescence and adulthood. All results are presented as the mean values ± SEM. *Asterisk* indicates significant between-group differences of the mean values (Student’s *t*-test for two independent groups, *p* < 0.05). **(A)** The body weight reduction in maternally deprived animals at weaning period, P35 and P60, was reduced compared with the controls (*p* < 0.05), while on P9 there was no statistically significant difference (*p* > 0.05). **(B)** The corticosterone levels were elevated in maternally deprived rats compared with the controls in adolescence (*p* < 0.05) and adulthood (*p* < 0.05).

To address whether maternal deprivation affected the animals’ development and growth, the animals were weighed upon separation from their mothers. At weaning, maternal deprivation caused a profound 56% loss of weight in stressed rats compared with the controls (Figure 1A). To address whether maternal deprivation affected the glucocorticoid levels in adolescent and adult animals, the concentration of circulating corticosterone was measured in the blood of the rats sacrificed on postnatal days 35 and 60 (P35 and P60, respectively). Maternally deprived animals exhibited 1.9- and 1.4-fold elevations in blood corticosterone levels on P35 and P60 (Figure 1B), respectively. This implies that the early life adversity caused profound phenotype changes connected with the early development arrest and the elevation of glucocorticoid levels at later stages of life, more so in adolescence than in adulthood.

**FIGURE 2:**
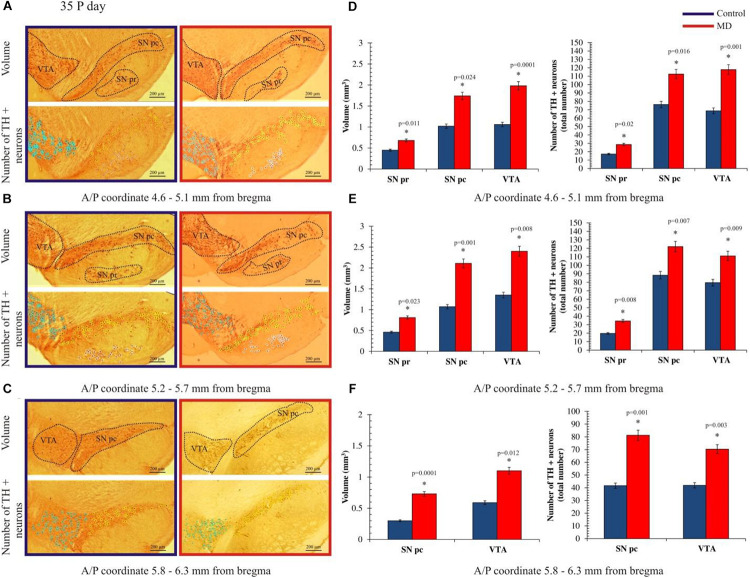
Effect of maternal deprivation on the volume of dopaminergic nuclei and the number of dopaminergic neurons in adolescence (postnatal day 35, P35). Representative examples of six sections are presented for **(A)** 4.6–5.1 mm from bregma; **(B)** 5.2–5.7 mm from bregma; and **(C)** 5.8–6.3 mm from bregma. The *upper pictures* depict surface areas, and these are marked with *dashed lines* encircling the dopaminergic structures, i.e., ventral tegmental area (VTA), substantia nigra pars compacta (SNpc), and substantia nigra pars reticulate (SNpr). The *lower pictures* depict the quantification of the number of tyrosine hydroxylase-positive (TH +) neurons, where TH + neurons belonging to the VTA, SNpc, and SNpr are shown are *purple*, *yellow*, and *white circles*, respectively. *Bar graphs* depict the comparisons between the maternally deprived and control rats for **(D)** 4.6–5.1 mm from bregma; **(E)** 5.2–5.7 mm from bregma; and **(F)** 5.8–6.3 mm from bregma. Volumes of the dopaminergic nuclei are presented on the *left*, while the total numbers of the TH + neurons per nucleus are represented on the *right*. The results are presented as the mean values ± SEM. *Asterisk* indicates significant between-group differences of the mean values (Student’s *t*-test for two independent groups, *p* < 0.05). ^∗^Increases in the volume of the dopaminergic nuclei and the number of TH + neurons in the maternally deprived (MD) group were present in all analyzed sections. Anterior/posterior (A/P) 4.60–5.10 mm caudally from bregma, there were 1.5- (*p* < 0.05), 1.7- (*p* < 0.05), and 1.9-fold (*p* < 0.001) increases in the volume of the dopaminergic nuclei and the 1.6- (*p* < 0.05), 1.5- (*p* < 0.05), and 1.7-fold (*p* < 0.05) increases in the number of TH + neurons in the SNpr, SNpc, and VTA. A/P 5.20–5.70 mm caudally from bregma, there were 1.8- (*p* < 0.05), 2.0- (*p* < 0.05), and 1.8-fold (*p* < 0.05) increases in the volume of the dopaminergic nuclei and 1.7- (*p* < 0.05), 1.4- (*p* < 0.05), and 1.4-fold (*p* < 0.05) increases in the number of TH + neurons in the SNpr, SNpc and VTA. A/P 5.80–6.30 mm caudally from bregma, there were 2.4- (*p* < 0.001) and 1.9-fold (*p* < 0.05) increases in the volume of the dopaminergic nuclei and 1.9- (*p* < 0.05) and 1.7-fold (*p* < 0.05) increases in the number of TH + neurons in the SNpr, SNpc, and VTA.

**FIGURE 3:**
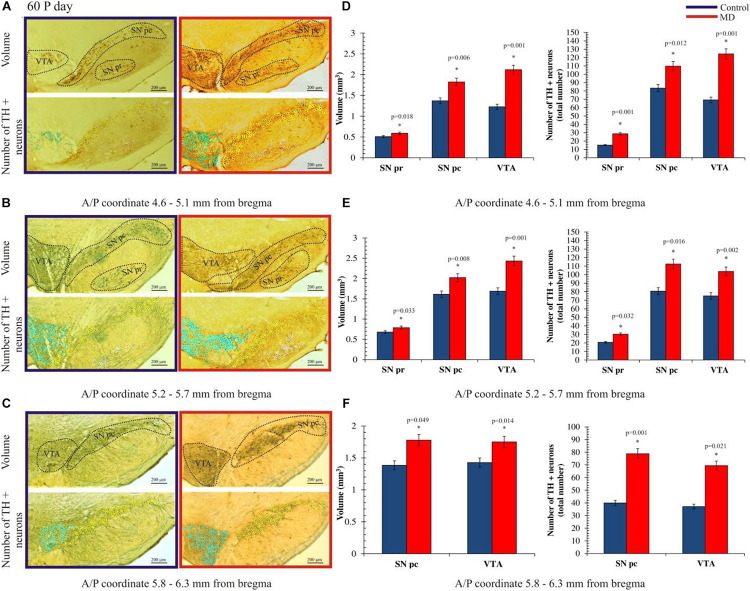
Effect of maternal deprivation on the volume of dopaminergic nuclei and the number of dopaminergic neurons in adulthood (postnatal day 60, P60). Representative examples of six sections are presented for **(A)** 4.6–5.1 mm from bregma; **(B)** 5.2–5.7 mm from bregma; and **(C)** 5.8–6.3 mm from bregma. The *upper pictures* depict surface areas, and these are marked with *dashed lines* encircling the dopaminergic structures i.e., ventral tegmental area (VTA), substantia nigra pars compacta (*SNpc*), and substantia nigra pars reticulate (*SNpr*). The *lower pictures* depict the quantification of the number of tyrosine hydroxylase-positive (TH +) neurons, where TH + neurons belonging to the VTA, SNpc, and SNpr are shown as *purple*, *yellow*, and *white circles*, respectively. *Bar graphs* depict the comparisons between the maternally deprived and control rats for **(D)** 4.6–5.1 mm from bregma; **(E)** 5.2–5.7 mm from bregma; and **(F)** 5.8–6.3 mm from bregma. Volumes of the dopaminergic nuclei are presented on the *left*, while the total numbers of the TH + neurons per nucleus are represented on the *right*. The results are presented as the mean values ± SEM. *Asterisk* indicates significant between-group differences of the mean values (Student’s *t*-test for two independent groups, *p* < 0.05). ^∗^Increases of the volume of the dopaminergic nuclei and the number of TH + neurons in the maternally deprived (MD) group were present in all analyzed sections. Anterior/posterior (A/P) 4.60–5.10 mm caudally from bregma, the increases in the volume of the dopaminergic nuclei were 1.2- (*p* < 0.05), 1.3- (*p* < 0.05), and 1.7-fold (*p* < 0.05), respectively, and those in the number of TH + neurons in the SNpr, SNpc, and VTA were 1.9- (*p* < 0.05), 1.3- (*p* < 0.05), and 1.8-fold (*p* < 0.05), respectively. A/P 5.20–5.70 mm caudally from bregma, increases in the volume of the dopaminergic nuclei were 1.2- (*p* < 0.05), 1.3- (*p* < 0.05), and 1.4-fold (*p* < 0.05), respectively, and those in the number of TH + neurons in the SNpr, SNpc, and VTA were 1.4- (*p* < 0.05), 1.4- (*p* < 0.05), and 1.4-fold (*p* < 0.05), respectively. A/P 5.80–6.30 mm caudally from bregma, there were 1.3- (*p* < 0.05) and 1.2-fold (*p* < 0.05) increases in the volume of the dopaminergic nuclei and 1.9- (*p* < 0.05) and 1.9-fold (*p* < 0.05) increases in the number of TH + neurons in the SNpc and VTA, respectively.

To address whether maternal deprivation affected the volume and composition of dopaminergic nuclei in adolescence and adulthood, histological slides containing coronal sections of ventral midbrain prepared from postmortem rat brains sacrificed on P35 and P60 were analyzed. The volume of the outlined nuclei was assessed for volumetric changes approximated by the surface area in three coordinates, whereas the number of tyrosine hydroxylase-positive (TH +) neurons was used to assess the changes in the number of dopaminergic neurons. On P35, 1.7-, 3. 1-, and 2.8-fold increases in the volume and 1.7-, 1.6-, and 1.6-fold increases in the number of TH + neurons were observed in SNpr, SNpc, and VTA, respectively (Figures 2A–F and Tables 1, 2). These increases were observed along the whole anteroposterior axis of the analyzed nuclei. On P60, 1.2-, 1.3-, and 1.7-fold increases in the volume of dopaminergic nuclei and 1.7-, 2.3-, and 2.6-fold increases in the number of TH + neurons were observed in SNpr, SNpc, and VTA, respectively (Figures 3A–F and Tables 1–3). These increases were observed along the whole anteroposterior axis of the analyzed nuclei. Altogether, early life adversity leads to a long-lasting pronounced increase in the volume of dopaminergic nuclei and the number of dopaminergic neurons among all major dopaminergic nuclei.

**TABLE 1:** Volumes of the substantia nigra pars reticulata (SNpr), substantia nigra pars compacta (SNpc), and ventral tegmental area (VTA) in adolescence (postnatal day 35, P35) and adulthood (postnatal day 60, P60).

A/P coordinate 4.60–5.10 mm from bregma		A/P coordinate 5.20–5.70 mm from bregma		A/P coordinate 5.80–6.30 mm from bregma	
		
Volume (mm^3^)		Volume (mm^3^)		Volume (mm^3^)	
		
	SNpr		SNpr		SNpr
*P35 day*		*P35 day*		*P35 day*	
Control	0.45 ± 0.03	Control	0.46 ± 0.02	Control	/
MD	**0.68 ± 0.06**	MD	**0.81 ± 0.12**	MD	/
*P60 day*		*P60 day*		*P60 day*	
Control	0.51 ± 0.01	Control	0.68 ± 0.04	Control	/
MD	**0.59 ± 0.02**	MD	**0.79 ± 0.01**	MD	/
	SNpc		SNpc		SNpc
*P35 day*		*P35 day*		*P35 day*	
Control	1.02 ± 0.05	Control	1.07 ± 0.07	Control	0.30 ± 0.01
MD	**1.74 ± 0.25**	MD	**2.11 ± 0.18**	MD	**0.73 ± 0.03**
*P60 day*		*P60 day*		*P60 day*	
Control	1.37 ± 0.03	Control	1.61 ± 0.08	Control	1.39 ± 0.08
MD	**1.83 ± 0.09**	MD	**2.02 ± 0.04**	MD	**1.78 ± 0.14**
	VTA		VTA		VTA
*P35 day*		*P35 day*		*P35 day*	
Control	1.06 ± 0.06	Control	1.35 ± 0.15	Control	0.59 ± 0.02
MD	**1.98 ± 0.13**	MD	**2.40 ± 0.26**	MD	**1.10 ± 0.16**
*P60 day*		*P60 day*		*P60 day*	
Control	1.23 ± 0.02	Control	1.69 ± 0.04	Control	1.43 ± 0.04
MD	**2.12 ± 0.11**	MD	**2.43 ± 0.10**	MD	**1.75 ± 0.08**

**All results are presented as mean values ± SEM. Numbers in bold are statistically significant mean values at p ≤ 0.05.**

**TABLE 2:** Number of TH + neurons in the substantia nigra pars reticulata (SNpr), substantia nigra pars compacta (SNpc), and ventral tegmental area (VTA) in adolescence (postnatal day 35, P35) and adulthood (postnatal day 60, P60).

A/P coordinate 4.60 – 5.10 mm from bregma		A/P coordinate 5.20 – 5.70 mm from bregma		A/P coordinate 5.80–6.30 mm from bregma	
		
Number of TH + neurons		Number of TH + neurons		Number of TH + neurons	
		
	SNpr		SNpr		SNpr
*P35 day*		*P35 day*		*P35 day*	
Control	17.2 ± 5.27	Control	19.7 ± 5.95	Control	/
MD	**28.6 ± 2.56**	MD	**34.4 ± 7.30**	MD	**/**
95% CI	11.4 ± 6.04	95% CI	14.7 ± 9.71	95% CI	/
*P60 day*		*P60 day*		*P60 day*	
Control	15.1 ± 3.86	Control	20.75 ± 3.97	Control	**/**
MD	**28.7 ± 2.69**	MD	**30.25 ± 5.58**	MD	**/**
95% CI	13.5 ± 4.85	95% CI	9.50 ± 7.06		
	SNpc		SNpc		SNpc
*P35 day*		*P35 day*		*P35 day*	
Control	76.2 ± 20.83	Control	88.3 ± 6.76	Control	41.60 ± 13.32
MD	**112.5 ± 15.81**	MD	**122.10 ± 16.25**	MD	**81.20 ± 8.40**
95% CI	36.3 ± 26.97	95% CI	33.8 ± 18.15	95% CI	39.6 ± 16.24
*P60 day*		*P60 day*		*P60 day*	
Control	83.50 ± 11.04	Control	80.75 ± 12.63	Control	40.00 ± 3.00
MD	**109.75 ± 9.60**	MD	**112.5 ± 14.50**	MD	**78.87 ± 6.21**
95% CI	26.25 ± 15.09	95% CI	31.75 ± 19.83	95% CI	38.87 ± 7.11
	VTA		VTA		VTA
*P35 day*		*P35 day*		*P35 day*	
Control	68.70 ± 17.95	Control	79.5 ± 10.25	Control	41.9 ± 3.15
MD	**117.8 ± 13.50**	MD	**111.10 ± 16.52**	MD	**70.30 ± 11.05**
95% CI	49.1 ± 23.16	95% CI	31.6 ± 20.05	95% CI	28.4 ± 11.85
*P60 day*		*P60 day*		*P60 day*	
Control	69.25 ± 8.87	Control	75.13 ± 4.71	Control	37.13 ± 5.94
MD	**124.38 ± 12.67**	MD	**103.75 ± 9.99**	MD	**69.50 ± 19.87**
95% CI	55.13 ± 15.95	95% CI	28.62 ± 11.39	95% CI	32.37 ± 21.39

**All results are presented as mean values ± SEM. Numbers in bold are statistically significant mean values at p ≤ 0.05;[Frame2] CI, confidence interval.**

**TABLE 3:** Density (number of TH + neurons per square millimeter) of the substantia nigra pars reticulata (SNpr), substantia nigra pars compacta (SNpc), and ventral tegmental area (VTA) in adolescence (postnatal day 35, P35) and adulthood (postnatal day 60, P60).

A/P coordinate 4.60–5.10 mm from bregma		A/P coordinate 5.20–5.70 mm from bregma		A/P coordinate 5.80–6.30 mm from bregma	
		
Density (Number of TH^+^neurons/mm^2^)		Density (Number of TH^+^neurons/mm^2^)		Density (Number of TH^+^neurons/mm^2^)	
		
	SNpr		SNpr		SNpr
*P35 day*		*P35 day*		*P35 day*	
Control	39 ± 5.79	Control	42 ± 4.53	Control	/
MD	43 ± 2.90	MD	44 ± 4.03	MD	**/**
*P60 day*		*P60 day*		*P60 day*	
Control	30 ± 3.94	Control	31 ± 2.85	Control	/
MD	**48 ± 3.10**	MD	**39 ± 3.80**	MD	**/**
	SNpc		SNpc		SNpc
*P35 day*		*P35 day*		*P35 day*	
Control	75 ± 9.50	Control	84 ± 5.10	Control	118 ± 3.2
MD	69 ± 8.30	MD	**59 ± 3.60**	MD	111 ± 2.8
*P60 day*		*P60 day*		*P60 day*	
Control	61 ± 3.40	Control	50 ± 2.10	Control	29 ± 2.60
MD	59 ± 3.70	MD	56 ± 4.60	MD	**46 ± 3.60**
	VTA		VTA		VTA
*P35 day*		*P35 day*		*P35 day*	
Control	65 ± 6.80	Control	62 ± 7.30	Control	71 ± 2.80
MD	61 ± 5.60	MD	**44 ± 2.50**	MD	67 ± 6.80
*P60 day*		*P60 day*		*P60 day*	
Control	56 ± 3.20	Control	45 ± 1.60	Control	26 ± 2.50
MD	51 ± 3.30	MD	50 ± 2.70	MD	**41 ± 7.10**

**All results are presented as mean values ± SEM. Numbers in bold are statistically significant mean values at p ≤ 0.05.**

## Discussion

The present study was focused on the effects of early life stress caused by maternal deprivation on the volume of dopaminergic nuclei and the number of dopaminergic cells within these nuclei. The key findings of this report are that both adolescent and adult brains are affected by early life adversity, which is accompanied by an elevation of corticosterone levels. Also, our study supports the findings of Hancock and Grant, who showed that after maternal separation in different periods of development, food intake dropped (Hancock and Grant, 2009). In particular, maternal deprivation increases the volumes of all dopaminergic nuclei and the total number of dopaminergic neurons therein in adolescence and adulthood.

Postnatal separation of pups from their mother caused an aberrant development of the dopaminergic system, which is postulated to play a role in the pathology of psychiatric diseases (Malnic et al., 1971). By restricting mother–pup contact, which also includes food deprivation at this stage, maternal deprivation disrupted the SHRP adaptation response and led to the abnormal functioning of the hypothalamic–pituitary–adrenal (HPA) axis and enhanced stress response (Champagne et al., 2009). Previous studies have shown that perinatal glucocorticoid treatment can permanently alter the basal and stress-induced levels of circulating glucocorticoids in experimental animals, and it is documented that endogenous adrenal steroids augment the activity of dopamine in the striatum (McArthur et al., 2005). Body weight loss in rats also corresponded with elevations of hypothalamic corticotropin-releasing hormone (CRH) messenger RNA (mRNA) expression (Kawaguchi et al., 2005) and increases in circulating adrenocorticotropic hormone (ACTH) (Wong et al., 1993) and corticosterone (Burden et al., 1993). The maternal deprivation method used in this study is different from that used in the majority of other studies reported in the literature, but the obtained results are in concordance with the outcome of a study conducted by Chocyk et al. (2011). They have shown a transient increase in the number of TH + neurons in SNpr in adolescent female rats as well as an increase in the number of TH + neurons in the VTA in adult female rats, with no changes in the volume of the examined structures. Depending on the stage development of the brain, there are several possible explanations for the increased number of TH-positive neurons in SN and VTA. Due to the dependence of the perikaryal TH protein levels on axonal transport rate, it is possible that TH is not present in the cell body or is present in large quantities (Nestler, 1992). Constitutive and sustained changes in the expression of TH + neurons in the midbrain are possible because, in the SN and VTA, Nurr1 and Pitx3 transcription factor expressions occur during the pre- and postnatal periods of brain development and are responsible for the survival of dopaminergic neurons (Ojeda et al., 2003; Korotkova et al., 2005; Katunar et al., 2009, 2010). There is also a possibility that the difference is caused by the TH expression from cells which are different from typical dopaminergic cells in SN and VTA (Ugrumov, 2009). Our results have shown an increase in the density of TH + neurons on P60 in both the examined stereotaxic range of SNpr as well as SNpc and VTA in the anterior/posterior (A/P) coordinate 5.80–6.30 mm from bregma. These results are in correlation with the study conducted on female Wistar rats obtained by Chocyk et al. (Chocyk et al., 2011) which showed that MD increased the number and density of TH + neurons in the SNpr, but not in the VTA, during adolescence. The results obtained in our study are similar to the study demonstrated by McArthur et al. (McArthur et al., 2007), which (in pharmacological terms) can imitate stress effects. De Souza et al. (de Souza et al., 2018) have shown that early life stress induced during the dark phase of the luminosity cycle increased the gene expressions of the dopaminergic receptors Drda and Drd2a. This effect was observed only in male rats.

Summing up, dopamine receptor expression is strongly regulated by aversive early life experiences. Actually, these effects may have a sex-specific developmental trajectory. It is known that MD disturbs the action of the HPA axis and affects the levels of circulating glucocorticoids (GCs) (de Kloet et al., 2005). GCs, acting *via* glucocorticoid and mineralocorticoid receptors and are widely expressed in the brain, affect the transcription of specific genes or exert many non-genomic effects. GCs have a substantial role in the regulation of brain development and maturation. Early life stress-induced imbalance in GC levels may program brain functions, including dopamine neurotransmission, for later life (de Kloet et al., 2005).

Finally, we cannot completely exclude the possibility that an increase in the total number of TH-positive neurons occurs due to neurogenesis at a certain point during ontogenesis, even though this concept is very disputable (Shan et al., 2006; Zhao and Janson Lang, 2009).

## Conclusion

This is the first study to provide evidence that early life stress caused by MD conducted on P9 in male rats leads to alterations in the morphological and biochemical parameters, including the body weight of rats, volume of the dopaminergic nuclei, number of TH + neurons in three different stereotaxic ranges through the overall rostro-caudal dimension, and the density of TH + neurons in the same ranges. The observed changes may facilitate the understanding of how early life stress may increase the tendency for neurological disorders, schizophrenia, and attention deficit hyperactivity disorder (ADHD). These disorders are known to be associated with irregular functions in dopaminergic neurotransmission, which may be mediated by aberrant maturation, which may include volumetric changes in the dopaminergic nuclei as well as alterations in the number and density of dopaminergic neurons.

## Data Availability Statement

All datasets presented in this study are included in the article/supplementary material.

## Ethics Statement

All experimental procedures were in compliance with EEC Directive (2010/63/EU) on the protection of animals used for experimental and other scientific purposes, and were approved by the Ethical Committee for the Use of Laboratory Animals of the School of Medicine, University of Belgrade (Approval No. 323-07-01245/2014-05/2).

## Author Contributions

All authors listed have made a substantial, direct and intellectual contribution to the work, and approved it for publication.

## Conflict of Interest

The authors declare that the research was conducted in the absence of any commercial or financial relationships that could be construed as a potential conflict of interest.
